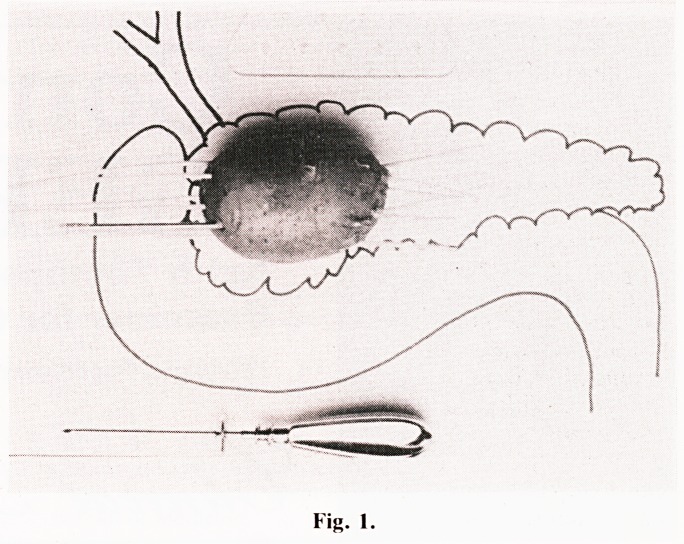# Carcinoma of the Pancreas and Selectron Brachytherapy

**Published:** 1990-03

**Authors:** Michael Golby, C. Giles Rowland

**Affiliations:** The Postgraduate Medical School, The Royal Devon and Exeter Hospital, Wonford, Exeter, England; The Postgraduate Medical School, The Royal Devon and Exeter Hospital, Wonford, Exeter, England


### Carcinoma of the Pancreas and Selectron Brachytherapy. A technique for possible cure or prolonged palliation.

**Michael Golby, ChM, FRCS and C. Giles Rowland, FRCR, DMRD, DMRT**
The Postgraduate Medical School, The Royal Devon and Exeter Hospital, Wonford, Exeter, England.

Carcinoma of the pancreas is now the fourth most common malignancy in many parts of the world. It is the major neoplasm in the hepato–biliary system which presents frequently as inoperable. It responds poorly to tele-radiotherapy and even bypass surgery gives the patient a very short time of relief. A search of the literature reveals the present dismal progress, even though the resection rate is rising and the operative mortality falling, results are still poor. *Trede* (1985) quotes resection as 21.3% of whom 28% survive 5 years for adeno carcinoma of the head of the pancreas. The resection rate of periampullary carcinoma is steady at 73% with a 50% 5 year survival. *Moossa* (1989) could not find a reported case of long term survival following resection of body and tail of the pancreas. The Japanese Pancreatic Association (*Tsuchiya* et al 1986) reviewed 106 cases of "small" carcinomas (i.e. under 2 cms. diameter) and found that 99% were resectable with a mortality of 3.9%. However, 56% of the total had already spread histologically and only 37% survived 5 years. In a series of 73 patients with irresectable carcinoma treated by bypass the median survival was only 7 months. (*McGrath* et al 1989). Our Exeter series is equally gloomy. Out of 74 patients who underwent a Whipple resection in a 10 year period only 3 survived 2 years and only 1 survived 5 years. This is in a community with over 38% of the population over 65 years of age. Early experience with pericavity neoplasms eventually led us to turn our attention to solid intra abdominal tumours particularly liver and pancreas. The encouraging work at King's College Hospital, London (*Nunneley 1989*) where an iridium-192 wire is passed down a biliary drain tube to treat cholangio-carcinoma has produced a mean overall survival of 16.8 months shows that tumours do respond to *brachytherapy*.

The low dose Caesium Selectron system gave us the concept that prostate tumours up 6 cms in diameter could be irradiated palliatively but it wasn't until the development of the 1.9 mm nylon catheter and the use of the high dose 10 curie Iridium sources coupled with the computer assisted programming of the new Selectron machine that we realised that we had the means of applying Brachytherapy to intra abdominal tumours. This was with the proviso that the tumours could be intubated in volumes of 8×4×4 cms. In theory there is no limit to the number of such lesions that can be treated, only the practical details of arranging the arrays of catheters.

### MATERIALS AND METHODS

In a 33 month period prior to 1988 100 patients were admitted to our hospital with a diagnosis of carcinoma of the pancreas, 22% were not operated upon, 58% had a bypass operation, one patient had her tumour resected and 19% were deemed to be untreatable at laparotomy. Further study of the notes of the bypass patients suggested that 87% had a large local tumour which could be 75% encompassed by one array of *HDR* Selectron catheters. During the same period of time several patients were operated upon for large (usually solitary) lesions of the liver or malignancy of the porta hepatis where the volume of the disease was less than 8×4×4 cms. At that time we did not envisage the use of more than one array of catheters in each patient but resolved in future to attempt adjunctive brachytherapy. Since then the technique has evolved.

At laporotomy through an upper inverted "V" incision the tumour mass is mobilised as much as possible. Superficial liver tumours are easy to manipulate but those deep within the right lobe may require ultrasound scanning to localise. I prefer to approach the head of the pancreas across the second part of the duodenum so that I can feel it between the finger and thumb of the left hand whilst passing the trocar. The necessary anastomoses are prepared but not sutured. To make the passage of the soft nylon catheters through the tumour feasible this is performed inside a trocar which is first passed by the surgeon with as much precision as possible to comply with the necessary grid pattern for radiation and to avoid major vessels (Fig. 1). The trocar is removed retrogradely. If the passage of the trocar or the catheter causes hemorrhage then that particular track is not used and its ends closed with catgut sutures. After this the edges of the tumour are marked radiologically with Cushing clips, the anastomoses completed and the abdomen closed with drainage and care not to kink the nylon catheters. It is sensible to encase these catheters in a large bore tube to stop damage, it also acts as a secondary wound drain. Post operative irradiation starts on the third day after CT scan and consists of 6 fractions of 5 Gys. to 85% isodose given over the next three days. All catheters are removed on the seventh day.

### RESULTS

Since February 1988 16 patients have been explored for malignant lesions of the liver, bilary tree and pancreas. In all cases pre-operative ultrasound or CT scanning have suggested localised disease. Two groups have resulted *i.e.* Group

**Fig. 1.**

1—Those catheterised for Selectron Brachytherapy, Group 2—those *not* catheterised for Selectron Brachytherapy.

### GROUP 1 TUMOURS CATHETERISED

1. B.T. F. 79 yrs. 17 × 12 × 10 cms 2nd. carcinoma L. lobe liver array 12 catheters 60% shrinkage painfree lived 12/52
2. R.S.B. M. 49 yrs. 6 cm. carcinoma head of pancreas Roux loop bypass array 6 catheters 3/12 later, alive and well 21 months later C.T. scan now normal
3. R.T. F. 71 yrs. 8 cms. carcinoma head of pancreas Roux loop bypass array 6 catheters died day 2 M.I.
4. P.M. F. 79 yrs. scirrhous carcinoma gall bladder and lower 1 cm of C.B.D. cholecystectomy explore C.B. D. sphincterotomy array 1 catheter down T. tube 1 week later, alive and well 11/12
5. L. deG. F. 40 yrs. 10 cms. carcinoma head of pancreas cholecysto-duodenostomy, gastro-jejunostomy 2/12 later array 9 catheters alive and well 7/12 later
6. R.F. F. 79 yrs. 6 cms. carcinoma head of pancreas Roux loop array 6 catheters died day 1 M.I. (bleed)
7. E.B. M. 75 yrs. 5 cms. papillary carcinoma of 2nd. part duodenum and head Roux loop array 6 catheters died day 6 haemorhage from duodenal ulcer and necrosis of pancreas
8. I.D. M. 78 yrs. perforated empyaemia of gall bladder C.B.D. explored tumour lower end array 1 catheter down T. tube 1 week later alive and well 2/12
9. M.T. F. 76 yrs. 6 cms. carcinoma body of pancreas irresectible, array 7 catheters alive at 12 weeks

### GROUP 2 TUMOURS NOT CATHETERISED

10. T.D. M. 60 yrs. ascites and continuous sheet of carcinoma from pancreas to liver untreatable died 6/52 later
11. J.M. M. 75 yrs. retroperitoneal mass from pancreas involving all structures untreatable died 2/52 later
12. V.W. F. 73 yrs. cholangiocarcinoma of porta hepatis and left hepatic duct Roux loop to rt. hepatic duct died 4/12 later *** in retrospect suitable for array 6 catheters 2 inside liver 4 along porta hepatis
13. A.P. M. 72 yrs. 6 cms. carcinoma head of pancreas choledocho-duodenostomy re-explored 9/12 later for obstructive jaundice Roux loop tumour 10 cms. considered too large *** in retrospect suitable died 5/12 later
14. J.M. F. 75 yrs carcinoma head of pancreas and gallstones cholecystectomy and explore C.B.D. frozen section histology negative patient declined 2nd. operation *** in retrospect suitable died 10/12 later
15. I.S. M. 68 yrs. 8.5 cms. lesion in rt lobe of large cirrhotic liver and multiple <.5 cms. satellite secondaries hepatic cell carcinoma *** suitable but unable to localise tumour no intra-operative ultrasound.
16. M.A. M. 74 yrs. cholecysto-jejunostomy and gastro-enterostomy for carcinoma of head of pancreas 2 yrs. later (after G.I. bleed) mass considered too large for brachytherapy, no response to external beam radiotherapy

Haemorrhgage has been encountered on three occassions and should be meticulouly controlled. It may have contributed to the death of patient 6.

Both the haemorrhage and pancreatic necrosis (?pancreatitis) in patient 7, were the natural progression of the disease not due to the intubation.

Most adenocarcinomas of the pancreas are fibrous and frequently frozen section histology is unhelpful this should not preclude intubation which can be performed on clinical diagnosis.

Transduodenal intubation seems preferrable to prevent possible pancreatic fistula formation which has not yet been encountered.

In retrospect the failure to use more than one array of catheters in tumours considered too large was wrong, even at the 50% isodose level brachytherapy has more to offer than external beam irradiation.

The impression was gained that many of the untreatable carcinomas of the pancreas with a sheet of retroperitoneal tumour had arisen from the body of the pancreas.

### DISCUSSION

Neoplasms of the upper right quadrant of the abdomen are commonly considered together since they often present to hospital late in the disease in frail, elderly patients who are acutely ill. Inspite of modern diagnostic aids it may not be possible to be certain of the primary site of malignancy prior to laporotomy. This is usually undertaken with a sense of doom that the lesion will be inoperable and that relief of symptoms will be short term. We have sought to demonstrate that there is now available an adjunctive form of brachytherapy for a large percentage of these patients which does not carry great risk of increased, early mortality whilst offering hope of prolonged palliation and even cure.

The technique can be applied at the time of major surgical intervention or up to three months later with hope of success and there is no reason why it could not be repeated after a longer period of time. Although to do so at less than one year interval would seem to be inappropriate with our current limited knowledge.

Future development of rigid catheters with a steerable tip may allow the treatment to be effected by interventionalist radiologists. Until that day arrives we believe the adoption of this treatment by more surgeons will lead to the prolonged survival of patients particularly with carcinoma of the head of the pancreas.

### REFERENCES

McGRATH, P. C., McNEIL, P. M., NEIFELD, J. F. *et al.* (1989) Management of biliary obstruction in patients with unresectable carcinoma of the pancreas. *Annals of Surgery* **209**, 284–288

MOOSSSA, A. R. (1989) Cancer of the bile ducts and pancreas. Saunders, London, 204

NUNNELEY, H. B., (1989) Cancer of the bile ducts and pancreas. Saunders, London, 94–97

TREDE, M. (1985) The surgical treatment of pancreatic carcinoma. *Surgery* **97**, 28–35

TSUCHIYA, R., TAKATOSH, N., HARADA, N., *et al.* (1986) Collective review of small carcinomas of the pancreas. *Annals of Surgery* **209**, 284–288

21:55:4416 Nov 1989

### NOTE

"Brachytherapy is radiotherapy given directly into a lesion as opposed to external beam, teletherapy.The Selectron HDR (high dose radiation) machine is produced by Nucletron. It has a system of remote after loading of a ten curie irridium 192 source attached to a one metre flexible cable. This can be loaded into an applicator (catheter) which has an external diameter of two millimetres. Computer programming and an array of up to 18 catheters can allow successful irradiation to a volume of tumour of 8 × 4 × 4 cms. in a few minutes. Several array of catheters can be used. There is also a low dose Selectron system (LDR) using caesium 137 where the delivery catheter is 8 mm. in diameter. Both these systems are of significant value because of their low capital cost and low running cost and also because of their adjunctive value in treatment of surgically inoperable neoplasms".

## Figures and Tables

**Fig. 1. f1:**